# Lack of generalization between explicit and implicit visuomotor learning

**DOI:** 10.1371/journal.pone.0224099

**Published:** 2019-10-17

**Authors:** Jinsung Wang, Shancheng Bao, Grant D. Tays

**Affiliations:** Department of Kinesiology, University of Wisconsin–Milwaukee, Milwaukee, Wisconsin, United States of America; Universidade Estadual Paulista Julio de Mesquita Filho, BRAZIL

## Abstract

Visuomotor adaptation has been thought to occur implicitly, although recent findings suggest that it involves both explicit and implicit processes. Here, we investigated generalization between an explicit condition, in which subjects reached toward imaginary targets under a veridical visuomotor condition, and an implicit condition, in which subjects reached toward visual targets under a 30-degree counterclockwise rotation condition. In experiment 1, two groups of healthy young adults first experienced either the explicit or the implicit condition, then the other condition. The third group experienced the explicit, then the implicit condition with an instruction that the same cognitive strategy could be used in both conditions. Results showed that initial explicit learning did not facilitate subsequent implicit learning, or vice versa, in the first two groups. Subjects in the third group performed better at the beginning of the implicit condition, but still had to adapt to the rotation gradually. In experiment 2, three additional subject groups were tested. One group experienced the explicit, then an implicit condition in which the rotation direction was opposite (30-degree clockwise rotation). Generalization between the conditions was still minimal in that group. Two other groups experienced either the explicit or implicit condition, then performed reaching movements without visual feedback. Those who experienced the explicit condition did not demonstrate aftereffects, while those who experienced the implicit condition did. Collectively, these findings suggest that visuomotor adaptation primarily involves implicit processes, and that explicit processes can add up in a complementary fashion as individuals become increasingly aware of the perturbation.

## Introduction

It is generally agreed that there are two types of motor learning: one that involves the acquisition of a novel motor skill, and the other that involves the adaptation of existing motor skills to novel sensorimotor conditions [1]. When one tries to learn a tennis overhead for the serve execution, for example, he attempts to improve his performance over a number of practice trials by updating his motor plan for the next trial based on the performance feedback that he received following the previous trial. In this case, the individual is *aware* of the task goal (i.e., to put the ball into the service box diagonally opposite) and also of the fact that the given skill is being acquired (i.e., the number of successful shots into the service box is increasing).

When existing motor skills are adapted, the involved neural processes can be somewhat different. For example, when one performs targeted reaching movements under a novel visuomotor condition in which the visual display of the reaching movements is rotated 30 degrees counterclockwise and she is not informed of this condition, she also attempts to improve her performance by updating her motor plan for the next trial based on the performance feedback from the previous trial. In this case, however, the task goal known to the individual often differs from that intended by the experimenter. That is, the task goal known to the individual is only to minimize the movement errors (which, to her, is more of a motor control, than learning, issue, given that she is not aware of the visuomotor perturbation), while the actual goal, only known to the experimenter, is for the subject to adapt to the rotated visual-display condition as fast as possible. In other words, the subject is *unaware* of the actual task goal (i.e., to adapt to the novel visuomotor condition) and also of the fact that she is adapting to the condition (i.e., her hand is moving straighter toward the position that is rotated 30 degrees clockwise from the visual target).

Apparently, the former example is a case of explicit learning (i.e., the subject is aware of what he is learning), and the latter a case of implicit learning (i.e., the subject is unaware of what she is learning). However, it is currently under debate whether the latter example indeed occurs implicitly or whether it also involves explicit learning. It has been previously thought that visuomotor adaptation occurs implicitly. Mazzoni and Krakauer demonstrated that when subjects were instructed explicitly how to deal with a visuomotor rotation, they were initially successful in negating the rotation; however, they failed to sustain explicit control and the movement errors became larger [2]. Benson et al. compared the pattern of visuomotor adaptation between the subjects who were informed of a visuomotor rotation condition prior to the experiment and those who were not, and revealed that while the former group of subjects reduced movement errors faster, they had increased trial-to-trial variability and longer reaction times [3]. They also showed smaller movement errors during catch trials and decreased aftereffects than the latter group. These findings have been viewed as evidence that visuomotor adaptation occurs implicitly, and that informing the subjects of the visuomotor perturbation can change the nature of visuomotor adaptation. More recently, however, this view has been challenged by the findings reported by Taylor and colleagues [4–7]. In these studies, the authors asked the subjects to provide a verbal report of their aiming direction on each trial while adapting to a visuomotor rotation, which allowed the authors to differentiate the explicit and implicit components that might underlie visuomotor adaptation [4–6]. The authors found that explicit and implicit learning processes were dissociable and followed distinctive time courses, based on which they concluded that visuomotor adaptation involves both explicit and implicit learning components.

Whereas the aforementioned task design allowed Taylor and colleagues to differentiate the explicit and implicit components successfully in their studies, the visuomotor adaptation task employed in their studies may be somewhat different from the typical visuomotor adaptation task employed in other previous studies. It is possible that the instruction given to the subjects (i.e., to report the aiming direction) in Taylor and colleagues’ studies caused the subjects to become more aware of the visuomotor rotation, which in turn caused them to make a more conscious effort to correct their movement based on the awareness of the rotation. In other words, the authors might have added an explicit component to a visuomotor adaptation task that otherwise might primarily involve an implicit component, by having the subjects verbally report their intended aiming direction. This is in line with an argument made by Leow et al. that requiring participants to report their aiming directions during visuomotor adaptation can affect the extent or persistence of implicit learning [8].

In the present study, we mainly attempted to determine the extent of generalization between two task conditions: one that mainly involved explicit learning, and the other that mainly involved implicit learning. In the former condition, subjects were asked to reach toward imaginary targets under a veridical visuomotor condition by using a cognitive strategy (i.e., mental rotation). It was assumed that this condition only involved explicit learning because the subjects were completely aware of the task goal (i.e., to reach toward the imaginary targets based on an instruction provided by the experimenters). In the latter, subjects were asked to reach toward visual targets under a novel visuomotor rotation condition, which is the typical condition employed in many previous studies in which subjects were not informed of the visuomotor perturbation [9–15]. Though it is possible that this condition may also involve explicit components, it was assumed that it mainly involved implicit learning because the subjects were unaware of the task goal (i.e., adapt to the novel visuomotor rotation while reaching toward visual targets). We have previously reported that when individuals were exposed to a 30-degree visuomotor rotation (which is the same rotation used in the present study), most of them were unaware of the rotation [16]. We hypothesized that if the two conditions had a reasonably large overlap in terms of their neural and/or cognitive processes (i.e., the latter condition also involves explicit components), initial visuomotor learning under one condition would facilitate subsequent visuomotor learning under the other condition to a great extent; if such facilitation were not observed, that would indicate that the overlap between the two conditions is minimal (i.e., the latter condition mainly involves implicit components).

In addition to the two aforementioned conditions, four other similar conditions were included and compared against each other in two separate experiments.

## Experiment 1

### Materials and methods

#### Participants

Twenty-seven neurologically intact young adults (age 22.3 ± 1.9 years old; 6 females), who were right handed and naïve to the experimental conditions employed in this study, participated in this experiment. Prior to participation, subjects signed an informed written consent, which was approved for this entire study by the Institutional Review Board of the University of Wisconsin–Milwaukee (Approval #16–163).

#### Apparatus

**Fig 1:**
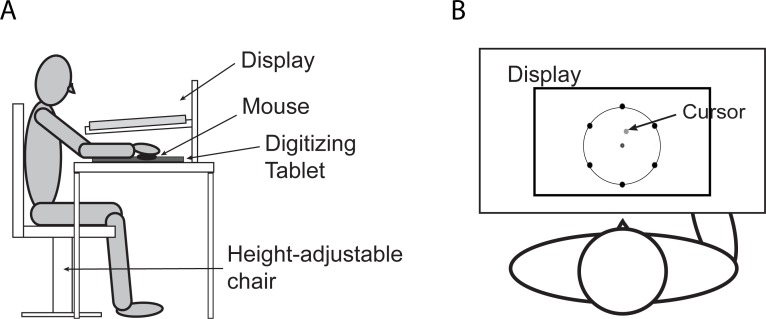
Experimental setup. (A) Subjects were seated on a height-adjustable chair placed in the front of the data acquisition device. They held a mouse in the hand and moved it to perform reaching movements on a digitizing tablet placed below the display. (B) In each movement trial, subjects made a reaching movement from start circle to one of six targets that appeared on the outer circle. Direct vision of the hand was blocked by the horizontal display; visual feedback was provided as a cursor indicating the mouse position on the tablet.

A data acquisition system that comprised a desktop PC (Dell Optiplex 9020, Round Rock, TX), a Wacom Intuos3 digitizing tablet (PTZ-1231W, Kazo, Japan; 12” x19”) and an LCD monitor (Dell S2415Hb; 24” diagonal) was used to collect data (Fig 1A). This system was incorporated with a computer program designed using Presentation® software (Neurobehavioral Systems, Inc., Berkeley, CA), which allowed us to record the subject’s reaching movements from a start circle (1.0 cm in diameter) to one of six targets (1.0 cm in diameter) that were pseudorandomly presented (Fig 1B). Subjects were seated on a chair facing a table, on which the data acquisition system was placed, with the right arm positioned on top of the digitizing tablet and under the horizontal display. Direct vision of the moving hand was blocked by the horizontal display; and a cursor representing the location of the center of a mouse (Wacom, ZC-100-02) held in the subject’s hand was provided to guide the subject’s reaching movement. The position of the mouse was sampled at 60Hz, low-pass filtered, and differentiated to yield resultant velocity values. Data were processed and analyzed using MATLAB (MathWorks, Natick, MA).

#### Experimental design

In general, subjects performed rapid reaching movements from a start circle to a target (10 cm away from the start circle) repeatedly with the right arm. They were instructed to move the cursor to the target rapidly and as straight as possible in response to the appearance of the target, stop without correcting their movement, and bring the cursor slowly to the start circle.

The experiment consisted of three sessions (separated by approx. 3 minutes): baseline (48 trials), initial learning (training; 120 trials) and subsequent learning (testing; 120 trials). In the *baseline* session, all subjects were familiarized with the general reaching movement. In the *training* session, subjects experienced either explicit or implicit conditions. In the explicit condition, subjects reached toward imaginary targets based on the following instruction: “A target will appear on the screen. Your task is to hit an imaginary target as accurately and straight as possible, by moving the cursor in the direction that is rotated 30 degrees *clockwise (CW)* from the visual target.” In the implicit condition, subjects reached toward visual targets based on the following instruction: “A target will appear on the screen. Your task is to hit the target as accurately and straight as possible.” In the former condition, the cursor was shown in a veridical condition; in the latter, the cursor was rotated 30 degrees *counterclockwise (CCW)* about the start position. In both conditions, targets were presented in a pseudorandom order (i.e., each target appeared once in 6 consecutive trials, called a ‘cycle’), and the cursor was always visible.

**Fig 2:**
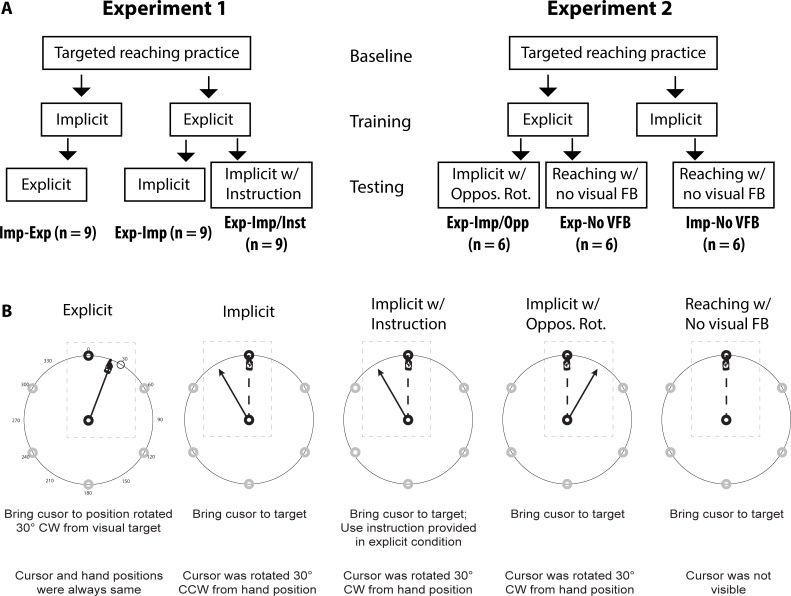
Characteristics of subject groups and conditions. (A) Conditions provided for each subject group during three experimental sessions. (B) Statements in first row describe task for each condition. In explicit condition, subjects reached toward imaginary targets that were rotated 30 degrees CW about start position from visible targets. In implicit condition, subjects reached toward targets under condition in which visual display was rotated 30 degrees CCW about start position. In implicit w/ instruction condition, subjects reached toward targets under condition in which visual display was rotated 30 degrees CCW about start position, and were told to use instruction provided during explicit learning. In implicit w/ Oppos. Rot. condition, subjects reached toward targets under condition in which visual display was rotated 30 degrees CW about start position. In reaching w/ no visual FB condition, subjects reached toward visible targets without receiving visual feedback of hand position. Statements in second row describe the cursor-hand mapping arrangement for each condition. Solid black arrows indicate cursor movement directions; broken black lines with hand sign at the end indicate hand movement directions. Landmark values (0–330) were only visible in explicit condition; the outer circle was visible in all conditions. Similar landmarks were also used by Taylor and colleagues [4, 5].

In the *testing* session, the subjects who experienced the implicit condition during the training session received the explicit condition (n = 9; called the “Imp-to-Exp” group). Among the subjects who experienced the explicit condition during the training session, a half of them received the implicit condition (n = 9; called the “Exp-to-Imp” group). The other half received the implicit condition, and also were given an instruction, prior to the beginning of this session, that the use of the instruction they received in the explicit condition would help their performance in this session as well (n = 9; called the “Exp-to-Imp/Inst” group). This group was included to determine whether the subjects would be able to learn the implicit condition better if they were told that the same instruction (i.e., reach toward a position that is rotated 30 degrees CW from the visual target) could be applied in both conditions. The arrangement of these conditions for each group is shown in Fig 2.

**Fig 3:**
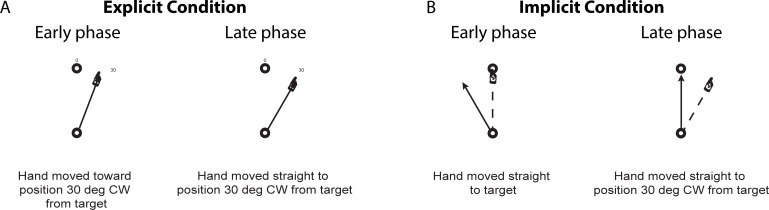
Experimental conditions. (A) In explicit condition, subjects were instructed to reach toward imaginary targets that were rotated 30 degrees CW about start circle from targets that appeared on outer circle. In early phase (left panel), subjects reached toward a position between visual and imaginary targets; in late phase (right panel), they reached accurately to imaginary target. Solid black lines with hand sign at the end indicate both cursor and hand movement directions. (B) In implicit condition, subjects were instructed to reach towards visual targets without knowing that the cursor was rotated 30 degrees CCW about the start circle. In early phase (left panel), subjects reached straight toward the target, while the cursor moved 30 degrees CCW from the target; in late phase (right panel), subjects moved the hand straight toward the positions that were rotated 30 degrees CW from the targets, which brought the cursor straight to the targets. Solid black arrows indicate cursor movement directions; solid black lines with hand sign at the end indicate hand movement directions.

The explicit and implicit conditions were designed in such a way that whereas the subjects’ performances would differ between the two conditions during the early phase of the training session, their performances in terms of controlling the direction of the *hand* movement would become the same by the end of the session (Fig 3). For example, when a target appeared at the position marked as “0”, the subjects in the *explicit* condition would move both the hand and the cursor toward a position marked as “30” at the early phase of the training session, but with some movement errors (see Fig 3A, left panel). By the end of the session, they would be able to move both the hand and the cursor straight to that imaginary target (see Fig 3A, right panel). In contrast, when a target appeared at the same position, the subjects in the *implicit* condition would first move their hand straight to that position, which would cause the cursor to move in the direction that was rotated 30 degrees CCW from that target position (see Fig 3B, left panel). By the end of the training session, they would move the hand straight to the position that was rotated 30 degrees CW from that target position, while bringing the cursor straight to the target (see Fig 3B, right panel). That is, in both conditions, the direction of hand movement would become the same by the end of the training session.

### Data analysis

To examine performance accuracy, we calculated initial direction error (DE), the angular difference between a vector from the start circle to the target and another vector from the hand position at movement start to that at peak arm velocity. DEs obtained in the explicit and implicit conditions were adjusted in such a way that the DE in the explicit and implicit conditions were subtracted by the mean DE across the last four cycles of the baseline session. In the explicit condition, DE was zero when the cursor moved perfectly straight to the imaginary targets; in the implicit condition, DE was zero when the cursor moved perfectly straight to the visual targets. (In both conditions, DE was zero, for example, when the target appeared in the 12 o’clock direction and the hand went straight in the 1 o’clock direction.) In both conditions, positive DE values indicated that the cursor was brought to a position that was on the right side (looking from the start circle) of the target; and negative DE values indicated that the cursor was brought to a position on the left side. The DE data were subjected to a repeated-measures ANOVA with group (Exp-to-Imp, Imp-to-Exp, Exp-to-Imp/Inst) and cycle (1–20) as a between- and a within-subject factor, respectively, which was performed for the explicit and the implicit conditions separately. The alpha level was set at 0.05 for the ANOVAs and also for post hoc between-group comparisons (Tukey’s tests).

### Results

**Fig 4:**
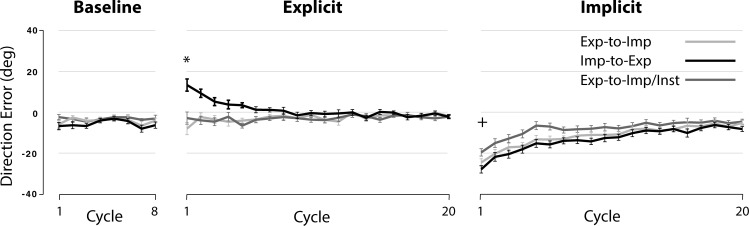
Changes in performance (mean ± SE) across cycles in Exp-to-Imp, Imp-to-Exp and Exp-to-Imp/Inst groups. Each data point represents mean DE across six consecutive trials (cycle). Performance changes across cycles for Exp-to-Imp, Imp-to-Exp and Exp-to-Imp/Inst groups during baseline (left), explicit (middle), and implicit (right) conditions are shown separately. * indicates significant difference between Imp-to-Exp and Exp-to-Imp groups and also between Imp-to-Exp and Exp-to-Imp/Inst groups, p < .05. + indicates significant difference between Imp-to-Exp and Exp-to-Imp/Inst groups, p < .05.

In this experiment, subjects in the Exp-to-Imp and Exp-to-Imp/Inst groups performed reaching movements under the explicit learning condition first, then under the implicit condition, while those in the Imp-to-Exp group did so under the implicit condition first, then under the explicit condition (see Fig 2). Fig 4 illustrates the changes in performance during the baseline, explicit learning and implicit learning sessions for each subject group. The repeated-measures ANOVA for the explicit condition indicated that the interaction effect of group and cycle was significant (F(16.65, 199.74) = 6.892, p < .001). Post hoc analyses revealed that the direction errors observed in the Imp-to-Exp group were significantly different from those observed in the other groups at the first cycle of the explicit condition (p < .05). In fact, the direction errors observed at this cycle were negative values in the Exp-to-Imp and Exp-to-Imp/Inst groups, whereas those were positive values in the Imp-to-Exp group. The positive errors reflect the aftereffects developed following initial learning under the implicit condition, which in turn indicates clearly that the visuomotor map of the subjects was altered following the implicit condition, although this change did not facilitate subsequent learning under the explicit condition in the Imp-to-Exp group. If it did, the performance of this group would have approximated the final adaptation level faster than that of the other groups; however, it was not the case.

The repeated-measures ANOVA for the implicit condition indicated a significant main effect of group (F(2, 24) = 4.22, p = .027) and cycle (F(12.69, 304.62) = 56.36, p < .001). The interaction effect between the two variables was not significant (p = .175). Post hoc analyses indicated that the difference between the Imp-to-Exp and the Exp-to-Imp groups at the first cycle of the implicit condition was not significant (p = .614), but the difference between the Imp-to-Exp and the Exp-to-Imp/Inst groups was (p = .023). This suggests that when adapting to the visuomotor rotation, the subjects in the Exp-to-Imp/Inst group were able to benefit from the instruction that was used during the explicit learning condition.

**Fig 5:**
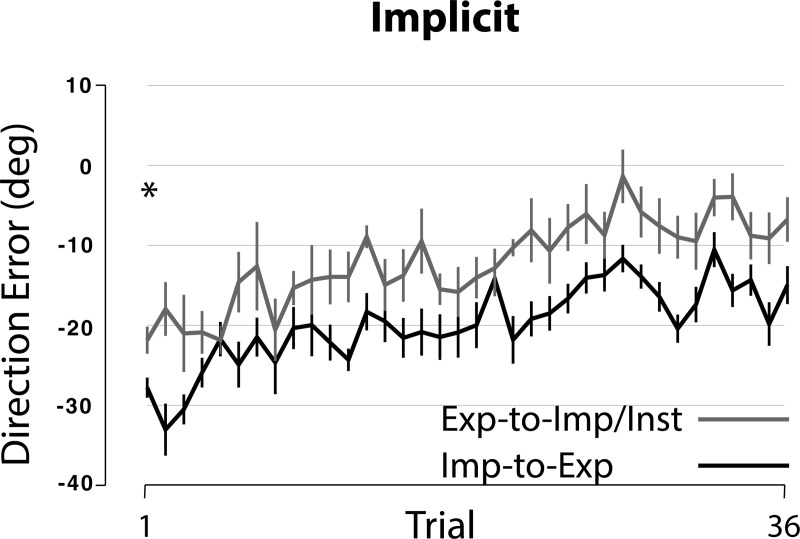
Changes in performance across trials in Imp-to-Exp and Exp-to-Imp/Inst groups. Each data point represents mean DE for a given trial. Performance changes across trials for Imp-to-Exp and Exp-to-Imp/Inst groups are shown separately. * indicates significant difference, p < .05.

Fig 5 depicts the changes in performance across *trials* during the implicit session for the Imp-to-Exp and Exp-to-Imp/Inst groups. A pairwise comparison between the two groups at trial 1 indicated a significant difference between the two groups (p = .014), suggesting that the subjects in the Exp-to-Imp/Inst group benefited from the instruction from the very first trial. However, the mean direction error at trial 1 was -27.7 (± 1.3 SE) and -21.7 (± 1.7 SE) degrees for the Imp-to-Exp and Exp-to-Imp/Inst groups, respectively; and the direction errors in the Exp-to-Imp/Inst group continued to decrease gradually. Given that the subjects already experienced the explicit condition and also that the difference between the two groups in terms of direction errors did not become smaller after the first trial, this result indicates that although the instruction had some beneficial effect, the effect of the prior explicit learning was minimal. (See more explanations in Discussion).

## Experiment 2

Findings from experiment 1 suggest that the extent of generalization between the explicit and implicit conditions was minimal, unless the subjects were told prior to the implicit condition that the cognitive strategy they used during the explicit condition would also be useful in the implicit condition. In experiment 2, we further investigated the effects of the explicit or implicit condition on varying types of testing sessions in three additional subject groups.

## Materials and methods

### Participants

Eighteen neurologically intact young adults (age 23.2 ± 2.4 years old; 6 females), who were right handed, participated in this experiment. All subjects signed an informed consent approved by the IRB.

#### Apparatus

The same apparatus used in experiment 1 was used in this experiment.

#### Experimental design

**Fig 6:**
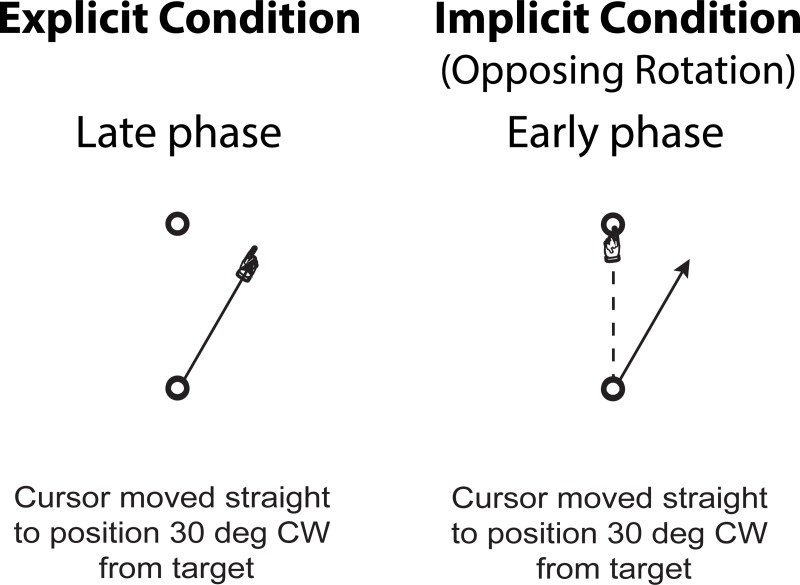
Experimental conditions for Exp-to-Imp/Opp group. In explicit condition, the same setup used in Experiment 1 was used. Solid black line with hand sign at the end indicates both cursor and hand movement directions. In implicit condition, subjects reached toward visual targets without knowing that the cursor was rotated 30 degrees CW about the start circle. In early phase, subjects reached straight toward the targets, while the cursor moved 30 degrees CW from the target. Solid black arrow indicates cursor movement direction; solid black line with hand sign at the end indicates hand movement direction.

As in experiment 1, experiment 2 also consisted of three sessions: baseline, training and testing. In the *baseline* session (48 trials), all subjects were familiarized with the general reaching movement. In the *training* session (120 trials), subjects experienced either the explicit or implicit condition (same as those employed in experiment 1). In the *testing* session (120 trials), two new conditions were introduced: implicit with an opposing rotation, and reaching without visual feedback. The former condition was the same as the implicit condition employed in experiment 1, except that the cursor was rotated 30 degrees *CW* about the start position. Adding this condition allowed us to investigate generalization between the explicit and implicit conditions in which the direction of the *cursor* movement (or the relationship between the target location and the cursor movement) during the late phase of the explicit condition was the same as that during the early phase of the implicit condition (Fig 6). (Cf. the direction of the *hand* movement was the same between the two conditions in experiment 1.) In the latter condition, subjects simply performed reaching movements without any visual feedback. This condition was included to determine whether learning under the explicit and implicit conditions would result in aftereffects.

In the testing session, the subjects who experienced the implicit condition during the training session received the reaching without visual feedback condition (n = 6; called the “Imp-to-NoVFB” group). Among the subjects who experienced the explicit condition during the training session, a half of them received the implicit with an opposing rotation condition (n = 6; called the “Exp-to-Imp/Opp” group); and the other half received the reaching without visual feedback condition (n = 6; called the “Exp-to-NoVFB” group).

#### Data analysis

**Fig 7:**
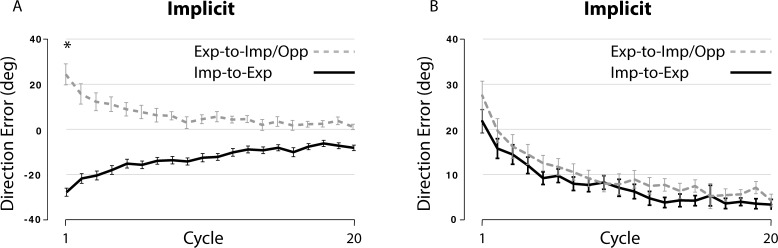
Changes in performance across cycles in Exp-to-Imp/Opp and Imp-to-Exp groups. (A) Each data point represents mean DE across six consecutive trials (cycle). Performance changes across cycles for Exp-to-Imp/Opp and Imp-to-Exp groups are shown separately. (B) Each data point represents baseline-adjusted DE in absolute values across six consecutive trials (cycle). * indicates significant difference, p < .05.

The DE data obtained from the three subject groups (Exp-to-Imp/Opp, Exp-to-NoVFB, Imp-to-NoVFB) tested in this experiment were investigated in the following ways:

(1) A comparison between the Imp-to-Exp and the Exp-to-Imp/Opp groups (implicit condition only). The DE data from the Imp-to-Exp group tested in experiment 1 were used for this comparison. Prior to the analysis, the DE data obtained from the implicit condition of each subject group were first adjusted in such a way that DEs in the implicit condition were subtracted by the mean DE of the last 3 cycles in the baseline session within each subject. Then, the baseline-adjusted DEs of the Imp-to-Exp group were converted to absolute values, which allowed a fair comparison between the data from the two subject groups. (As you would see in the Results section (Fig 7A), the original DE data from the Imp-to-Exp group were all in negative values, while those from the Exp-to-Imp/Opp group were all in positive values.) Finally, these baseline-adjusted DE data, in absolute values, from the implicit condition of the two subject groups were subjected to a repeated-measures ANOVA (Imp-to-Exp, Exp-to-Imp/Opp) with group and cycle (1–20) as a between- and a within-subject factor, respectively.

(2) A comparison between the Exp-to-NoVFB and the Imp-to-NoVFB groups. The DE data from the testing session of the two subject groups were subjected to a repeated-measures ANOVA with group (Exp-to-NoVFB, Imp-to-NoVFB) and cycle (1 and 2) as a between- and a within-subject factor, respectively.

The alpha level was set at 0.05 for the ANOVAs and also for post hoc comparisons.

### Results

(1) A comparison between the Imp-to-Exp and the Exp-to-Imp/Opp groups.

The changes in performance across cycles, using the original DE data, during the implicit condition for the Imp-to-Exp and Exp-to-Imp/Opp groups are shown in Fig 7A. DEs at cycle 1 were substantially different between the two subject groups in that they were similar in size, but opposite in terms of the sign. Fig 7B illustrates those changes using the baseline-adjusted DEs in absolute values, which were similar between the two subject groups. The repeated-measures ANOVA indicated a significant main effect of cycle (F(19, 247) = 42.09, p < .001). However, neither the main effect of group nor the interaction effect between the two variables was significant (p = .211 and .733, respectively). These data indicate that initial learning under the explicit condition did not facilitate subsequent learning under the implicit condition in the Exp-to-Imp/Opp group.

(2) A comparison between the Exp-to-NoVFB and the Imp-to-NoVFB groups.

**Fig 8:**
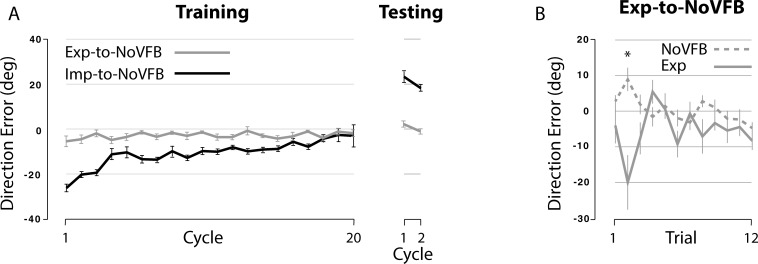
Changes in performance in Exp-to-NoVFB and Imp-to-NoVFB groups. (A) Each data point represents mean DE across six consecutive trials (cycle). Performance changes across cycles in Exp-to-NoVFB and Imp-to-NoVFB groups are shown for training (left) and testing (right) sessions separately. (B) Each data point represents DE for a given trial. Performance changes across trials in Exp-to-NoVFB group are shown for training (Exp) and testing (NoVFB) sessions separately. * indicates significant difference, p < .05.

Fig 8A illustrates the changes in performance across cycles during the training (left) and testing (right) sessions for the two subject groups separately. The repeated-measures ANOVA using the data from the testing session indicated that the main of group was significant (F(1,10) = 90.77, p < .001). The main effect of cycle or the interaction effect between the two variables was not significant (p = .561 and .249, respectively). DEs were significantly different between the two subject groups at both cycles 1 and 2 (p < .05). These data indicate that the implicit condition resulted in aftereffects, although the explicit condition didn’t.

We also plotted a graph depicting the changes in performance across trials (1–12) during the training and testing sessions within the Exp-to-NoVFB, shown in Fig 8B, because the direction errors at cycle 1 appeared somewhat different between the two sessions. Pairwise comparisons of the trial data (trials 1–16) indicate that DEs were not significantly different between the two sessions (except at trial 2), which confirms that the explicit learning condition did not result in aftereffects.

## Discussion

The results from experiment 1 indicate that initial learning under the implicit condition did not facilitate subsequent learning under the explicit condition in the Imp-to-Exp group, as indicated by large direction errors with the opposite sign at the beginning of the explicit learning condition. If initial learning under the implicit condition had a facilitative effect, the direction errors at the beginning of the explicit condition would have been negative and closer to zero. Likewise, initial learning under the explicit condition had no influence in subsequent learning under the implicit condition in the Exp-to-Imp group either, as indicated by the lack of difference between the two groups. These findings indicate that the extent of overlap (in terms of neural or cognitive processes) must be minimal between the explicit and implicit conditions employed in this study.

It should be noted that a neural imaging study has demonstrated overlapping brain activation between a mental rotation task and a visuomotor adaptation task (equivalent to our explicit and implicit conditions, respectively) in the prefrontal regions (right dorsolateral prefrontal cortex and the bilateral inferior parietal lobules) [17]. Here, the overlap was only observed during the early, and not late, phase of visuomotor adaptation, indicating that the overlap is not very large. Based on that finding, the authors suggested that spatial working memory processes underlying mental rotation are also involved during the early stage of visuomotor adaptation. This does not necessarily mean that the spatial working memory processes engaged during visuomotor adaptation have to do with knowing the visuomotor perturbation explicitly. Those processes may rather reflect the cognitive processes engaged as the subjects tried to deal with the *spatial* errors (i.e., largely curved hand-paths caused by the perturbation). This interpretation is supported by our finding (i.e., lack of generalization between the explicit and implicit conditions); and thus, the extent of overlap in terms of *explicit* processes between the explicit and implicit conditions employed in this study also seems to be minimal.

The results from the Exp-to-Imp/Inst group further support this argument (i.e., the extent of overlap between the two conditions is minimal). The first piece of evidence comes from the comparisons made among the Exp-to-Imp, Imp-to-Exp and Exp-to-Imp/Inst groups. When the subjects in the Exp-to-Imp/Inst group were told that the use of the instruction provided for the explicit condition would help them perform better during the next session, they adapted to the rotation better than those in the Imp-to-Exp group. As depicted in Fig 4 (right panel, implicit condition) and 5, however, the difference between the two groups is attributed to the fact that the subjects in the former group adapted better only by reducing the direction error at the beginning of the implicit condition. After that, the direction errors were gradually reduced both in terms of cycles (from cycle 1 to cycle 5) and trials (from trial 1 to trial 27). This pattern of performance change is quite different from that observed during the explicit condition in the Exp-to-NoVFB group (Fig 8B, solid line). That is, during that condition, the direction errors were not gradually reduced, but rather varied substantially from trial to trial. These results suggest that the beneficial effect of the instruction only affected the beginning stage of learning (i.e., reducing the initial error by approx. 6 degrees at trial 1) in the Exp-to-Imp/Inst group, and that the subjects in this group still had to adapt gradually to the visuomotor rotation in the following trials/cycles.

This interpretation is supported by a finding reported in Benson et al.’s study, in which the pattern of visuomotor adaptation was compared between a group of subjects who were given prior to adaptation an instruction as to how to deal with a perturbation (30-deg. rotation) and those who were not [3]. In that study, the difference between the two groups in terms of direction errors at trial 1 was approximately 11 degrees (see Fig 3A in that study); and the errors in the former group gradually decreased in the next 30 trials or so. In fact, the beneficial effect of the instruction was smaller in our study, given that the difference between the Imp-to-Exp and Exp-to-Imp/Inst groups was smaller (i.e., 6 degrees at trial 1) even after the subjects in the Exp-to-Imp/Inst group already experienced the explicit learning condition. These findings suggest that the effect of initial learning under the explicit condition on subsequent learning under the implicit condition (esp. without considering the benefit of the instruction per se) was also minimal in the Exp-to-Imp/Inst group.

Another piece of evidence comes from the comparison between the Imp-to-Exp and the Exp-to-Imp/Opp groups. This condition was tested to determine whether better generalization would occur if the direction of the cursor, as opposed to the hand, movement were the same between the explicit and implicit conditions. By rotating the visual display 30 degrees CW, the direction of the cursor movement during the late phase of the explicit condition became the same as that during the early phase of the implicit condition. Yet, the extent of generalization did not increase at all. Finally, the results from the Exp-to-NoVFB and Imp-to-NoVFB groups also provide support, by demonstrating that visuomotor learning under the implicit condition resulted in large aftereffects whereas visuomotor learning under the explicit condition did not (see Fig 8). This is consistent with a previous report which demonstrated that when subjects were instructed to aim toward a position that was rotated 45 degrees CW relative to a visual target (i.e., equivalent to the explicit condition in the present study), they hit the target successfully within the first few trials [2]. When they switched back to the baseline condition, however, there was no aftereffect. Given that the presence of aftereffects is typically considered as evidence that an internal model was developed as a result of motor adaptation [13,18–22], these findings indicate that the neural processes underlying the two conditions differ from each other.

Collectively, these findings suggest that the extent of overlap between the explicit and implicit learning conditions employed in this study is minimal; and they support the idea that “typical” visuomotor adaptation, which occurs without the subject’s awareness of the visuomotor perturbation, mainly involves implicit, but not explicit, components. Here, it should be noted that the subject’s awareness of the visuomotor perturbation can be influenced by several factors, such as the size of the rotation (e.g., 75-deg. [22–24] vs. 45-deg. [2, 4, 25] vs. 30-deg. [3, 13, 15, 16] rotation) and whether the perturbation is provided gradually or abruptly (which in turn can influence the size of aftereffects [2, 20, 22]). The awareness of the perturbation can also vary across individuals. In our previous study, for example, a post-experiment interview confirmed that most subjects who experienced a 30-degree rotation from the beginning of the adaptation session thought that “something” happened, although a few knew that the visual display was rotated [16]. This points to the possibility that individuals may be aware of the given visuomotor perturbation to a certain degree, based on which the involvement of explicit components will be determined (i.e., the more aware of the perturbation, the more explicit components will be involved). When both implicit and explicit components are available during visuomotor adaptation, it is likely that the two components contribute to the adaptation differentially, but in a complementary fashion, such that the explicit components may allow faster learning (cf. [2, 3]), while the implicit components may help the obtained motor memory to last longer (e.g., larger and longer-lasting aftereffects cf. [8, 20, 26]). Thus, for example, when an individual learns a skill that requires visuomotor adaptation (e.g., endoscopic surgery), combining repetitive practice (probably resulting in both implicit and explicit components) with specific instructions (increasing the portion of explicit components) may lead to an optimal learning experience.

In conclusion, the present study demonstrates that the extent of generalization between an explicit and an implicit visuomotor learning condition is minimal. This finding provides support to the traditional view that the “typical” visuomotor adaptation task primarily involves implicit learning components [2, 3], and further suggests that explicit components can add up in a complementary fashion to the implicit components based on the individual’s awareness of the visuomotor perturbation. A recent finding reported by Neville and Cressman [27] provides some support to this argument by demonstrating that two factors that influenced the subject’s awareness of visuomotor perturbations (i.e., provision of a cognitive strategy, the size of rotation) interacted with each other to vary the pattern of visuomotor adaptation in healthy young adults. Our concluding argument, however, may need to be taken with caution, given that the findings reported in the present study are based on the data from a relatively small sample size (9 participants in 3 groups, 6 participants in 3 groups). Further research with a larger sample size is warranted to determine more systematically the role of the individual’s awareness, which can be influenced by multiple factors (e.g., the size and the presentation schedule of a visuomotor perturbation, the provision of an instruction, requiring to report aiming directions) in altering the nature of visuomotor adaptation.
